# Immunohistochemistry of Programmed Cell Death in Archival Human Pathology Specimens

**DOI:** 10.3390/cells1020074

**Published:** 2012-05-07

**Authors:** Kazuhisa Hasui, Taku Nagai, Jia Wang, Xinshan Jia, Katsuyuki Aozasa, Shuji Izumo, Yoshifumi Kawano, Takuro Kanekura, Yoshito Eizuru, Takami Matsuyama

**Affiliations:** 1 Division of Immunology, Department of Infection and Immunity, Institute Research Center (Health Research Course), Kagoshima University Graduate School of Medical and Dental Sciences, Sakuragaoka 8-35-1, Kagoshima 890-8544, Japan; Email: tanaga@m.kufm.kagoshima-u.ac.jp (T.N.); wangjia0829jp@yahoo.co.jp (J.W.); matuyama@m.kufm.kagoshima-u.ac.jp (T.M.); 2 Department of Pathology, China Medical University, 92 Bei Er Ma Lu, He Ping Qu, Shenyang 110001, China; Email: kaxschina@yahoo.co.jp; 3 Department of Pathology, Graduate School of Medicine, Faculty of Medicine, Osaka University, Yamadaoka 565-0871, Suita, Japan; Email: aozasa@molpath.med.osaka-u.ac.jp; 4 Chronic Viral Diseases Division of Molecular Pathology, Center for Chronic Viral Diseases (Infection and Immunity), Institute Research Center (Health Research Course), Kagoshima University Graduate School of Medical and Dental Sciences, Sakuragaoka 8-35-1, Kagoshima 890-8544, Japan; Email: izumo@m.kufm.kagoshima-u.ac.jp; 5 Division of Pediatrics, Department of Developmental Medicine, Institute Research Center (Heath Research Course), Kagoshima University Graduate School of Medical and Dental Sciences, Sakuragaoka 8-35-1, Kagoshima 890-8544, Japan; Email: ykawano@m3.kufm.kagoshima-u.ac.jp; 6 Division of Dermatology, Department of Sensory Organology, Institute Research Center (Advanced Therapeutics Course), Kagoshima University Graduate School of Medical and Dental Sciences, Sakuragaoka 8-35-1, Kagoshima 890-8544, Japan; Email: takurok@m2.kufm.kagoshima-u.ac.jp; 7 Chronic Viral Diseases Division of Persistent & Oncogenic Viruses, Center for Chronic Viral Diseases (Infection and Immunity), Institute Research Center (Health Research Course), Kagoshima University Graduate School of Medical and Dental Sciences, Sakuragaoka 8-35-1, Kagoshima 890-8544, Japan; Email: ccvdgan@m.kufm.kagoshima-u.ac.jp

**Keywords:** antigen retrieval and immunohistochemistry, programmed cell death, apoptosis, autophagy, archival formalin-fixed and paraffin-embedded human pathology specimen

## Abstract

Immunohistochemistry (IHC) for detecting key signal molecules involved in programmed cell death (PCD) in archival human pathology specimens is fairly well established. Detection of cleaved caspase-3 in lymphocytes in rheumatoid arthritis (RA) and gastric surface foveolar glandular epithelia but not in synoviocytes in RA, gastric fundic glandular epithelia, or nasal NK/T-cell lymphoma (NKTCL) cells suggests anti-apoptotic mechanisms in cell differentiation and in oncogenesis such as the induction of survivin. Enzymatically pretreated and ultra-super sensitive detection of beclin-1 in synoviocytes in RA and gastric fundic glandular epithelia suggests enhanced autophagy. The deposition of beclin-1 in fibrinoid necrosis in RA and expression of beclin-1 in detached gastric fundic glandular cells suggest that enhanced autophagy undergoes autophagic cell death (ACD). NKTCL exhibited enhanced autophagy through LC3 labeling and showed densely LC3 labeled cell-debris in regions of peculiar necrosis without deposition of beclin-1, indicating massive ACD in NKTCL and the alternative pathway enhancing autophagy following autophagic vesicle nucleation. Autophagy progression was monitored by labeling aggregated mitochondria and cathepsin D. The cell-debris in massive ACD in NKTCL were positive for 8-hydroxydeoxyguanosine, suggesting DNA oxidation occurred in ACD. Immunohistochemical autophagy and PCD analysis in archival human pathology specimens may offer new insights into autophagy in humans.

## Abbreviations

AR: antigen retrieval
IHC: immunohistochemistry
PDC: programmed cell death
ACD: autophagic cell death
RA: rheumatoid arthritis
HP: *Helicobacter pylori*
EBV: Epstein-Barr virus
NK: natural killer
synoviocytes: synovial fibroblasts
EDTA: ethylendiaminetetraacetic acid
HRP: horse radish peroxidase
CSA: catalyzed signal amplification
nsCSA system: new simplified CSA system
Atg: autophagy-related protein
Vps: vascular protein sorting-associated protein
Vps15: serine/threonine-protein kinase Vps15
Vps34: phosphatylinositol 3-kinase
Flip: Flice (caspase-8)-like inhibitory protein
TIA-1: T-cell restricted intracellular antigen-1
Bcl-2: B-cell lymphoma 2
LC3: mammalian Atg8 homologue light chain 3
8-OHdG: 8-hydroxydeoxyguanosine
TG: thymidine glycol
iNOS: inducible nitric oxide synthase
NG: nitroguanosine
NKTCL: NK/T-cell lymphomas
EBER-1: EBV-encoded small RNA-1
ROS: reactive oxygen species
Bcl-X: Bcl-2-like 1

## 1. Introduction

Recent molecular and cell biological research efforts have been rewarded by fruitful results and many antibodies against signal transduction molecules and their antagonists have been supplied commercially. Through developed antigen retrieval and immunohistochemistry (AR-IHC) using these antibodies, it becomes possible to label these signal transduction molecules and antagonists in archival formalin-fixed and paraffin-embedded human pathology specimens, a huge number of which have been collected in the so-called Department of Human Pathology. Comparative AR-IHC using antibodies against the representative signal transduction molecules and antagonists in the archival human pathology specimens with and without various diseases is expected to be informative about differences in features of signal transduction between normal and diseased human cells in the tissues and to contribute to the medicine for the diseases and probably to further molecular and cell biological research.

Apoptosis, a form of programmed cell death (PCD) [1,2], has been defined as PCD type I, and is dependent on caspases [3]. AR-IHC using the well known anti-cleaved caspase-3 antibody (Table 1) has recently been introduced in the archival human pathology specimens [4].

Molecular mechanisms of autophagy and autophagic cell death (ACD), known as type II PCD [5,6,7,8], have been clarified [7,9,10,11,12,13]. Well known antibodies against beclin-1, which forms a complex with Atg14, Vps34, and Vps15 for autophagic vesicle nucleation, and against LC3 (LC3-II), which localizes in the membrane of autophagosomes, are supplied commercially (Table 1). Using these antibodies, we performed AR-IHC on archival human pathology specimens for detecting autophagy [14,15,16].

In the present report, we aimed to differentiate apoptosis, autophagic cell death (ACD) and other types of PCD using archival human pathology specimens. We describe our investigations involving AR-HC detecting apoptosis and autophagy in inflamed hyperplastic and degenerative lesions in rheumatoid arthritis (RA). This paper also reports on homeostatic mass control in the gastric fundic glandular mucosa with and without *Helicobacter pylori* (HP) [14] and peculiar necrosis in Epstein-Barr virus (EBV)-related nasal natural killer (NK)/T-cell lymphoma [15,16], and includes a discussion on the applications of AR-IHC for PCD in human pathology and autophagy research in humans.

**Table 1:** Antibodies and their immunohistochemical detection methods.

Antibody	Source	Dilution of Antibody	Immunohistochemistry	
			Antigen Retrieval (AR)	Detection Method (IHC)
Cleaved caspase-3	5A1, Asp175, Cell Signaling Co.	1:200	Heating-AR* or Heating-AR**	Ordinary IHC
Beclin-1 (H-300)	SC-11427, Santa Cruz Biotech. Inc.	1:50	Enzymatic-AR	Ultra-super sensitive IHC
LC3	PM036/MBL.	1:1000	Heating-AR**	Ordinary IHC
Flice (caspase-8) inhibitory protein (Flip)	ab4042/Abcam.	1:50	Heating-AR**	Ordinary IHC
TIA1	TIA1/Coulter Immunology.	1:500	Heating-AR	Ordinary IHC
Bcl-2	M0887/Dako.	1:100	Heating-AR	Ordinary IHC
AE-1 (mitochondria)	AE-1/Leinco Technologie Inc.	1:50	Heating-AR**	Ordinary IHC
Cathepsin D	NCL-CDm (C5)/Vision Biosystems.	1:100	Heating-AR**	Ordinary IHC
8-hydroxydeoxy- guanosine (8-OHdG)	N45.1, Japan Institute for the Control of Aging.	1:200	Heating-AR**	Ordinary IHC*
thymidine glycol (TG)	2E8, Japan Institute for the Control of Aging.	1:200	Heating-AR**	Ordinary IHC*
Inducible nitric oxide synthase (iNOS)	SC-651, Santa Cruz Biotech. Inc.	1:100	Heating-AR	Ordinary IHC
Nitroguanosine (NG)	NO2G52, Dujindo Laboratories.	1:100	Heating-AR**	Ordinary IHC
***Antigen retrieval (AR)*** Heating-AR: Deparaffinized sections are heated in 0.01 M citrate buffer pH 6 (S2031, ChemMate, Target Retrieval Solution, Dako) for 5 min at 120 °C by autoclaving. Heating-AR*: Deparaffinized sections are heated in EDTA solution pH > 9 (S3307, Antigen Retrieval Reagent, high pH, Dako) for 5 min at 120 °C by autoclaving. Heating-AR**: Deparaffinized sections are heated in citrate buffer (Diva Decloaker, Biocare Medical) at 120 °C by autoclaving independently from the AR solution pH. Enzymatic-AR: Deparaffinized sections are treated with 200 mg/mL proteinase K Tris buffer saline solution for 10 min at room temperature. ***Detection method (IHC)*** Ordinary-IHC: Super sensitive IHC (horse radish peroxidase (HRP)-labeled and secondary antibody-labeled polymer reagent method, such as ChemMate Envision system (K5027, Dako). Ordinary-IHC*: Super sensitive IHC (alkaline phosphatase (AlP)-labeled and secondary antibody-labeled polymer reagent method, such as Histofine Simple Stain AP(M), Nichirei BioSci. Inc.). Ultra-super sensitive IHC: IHC comprising HRP-labeled and secondary antibody-labeled polymer reagent method with catalyzed reporter deposition (CARD) reaction and visualizing deposition via the HRP-labeled streptavidin method, enabling suppression of non-specific reaction of the primary and secondary antibodies and of diffusion of deposition in the CARD reaction.				

## 2. Inflamed Hyperplastic and Degenerative Lesions in RA

Synovial tissue in RA exhibits hyperplastic and degenerative synovial cells along with fibrinoid necrosis and chronic inflammatory cell infiltration with lymph follicle formation. Synovial fibroblasts (synoviocytes) and macrophages activate molecular mechanisms against apoptosis [17]; their degeneration is believed to be due to a mechanism other than apoptosis.

**Figure 1:**
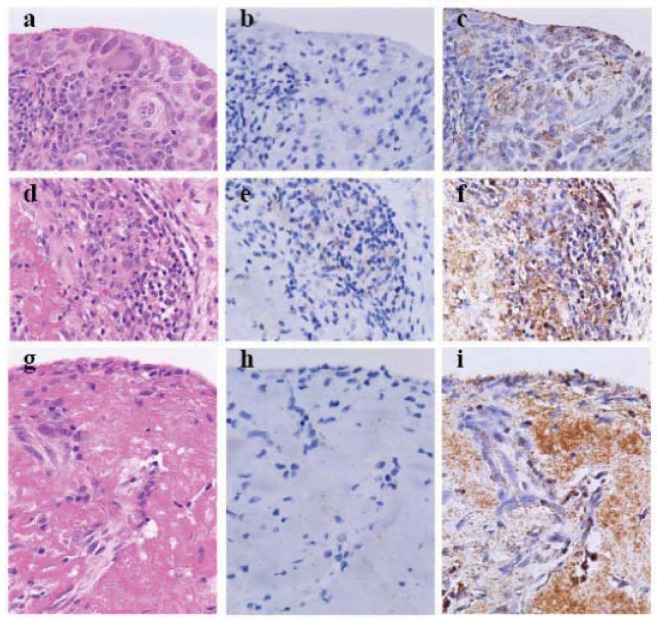
Cleaved caspase-3 and beclin-1 in the synovial tissue with rheumatoid arthritis (×40, Olympus BX50, FUJIFILM HC-300) (**a–c**) Thickened, inflamed and slightly degenerated synovial tissue. (**d–f**) Fibrinoid degeneration of synovial tissue with aggregated lymphocytes. (**g–i**) Fibrinoid degeneration of synovial tissue. (**a d and g**) Heamatoxylin-eosin staining (HE). (**b, e and h**) Heating antigen retrieval and super sensitive immunohistochemistry of cleaved caspase-3. (**c, f and i**) Enzymatic AR and ultra-super sensitive IHC of beclin-1. In rheumatoid arthritis synovial tissue synoviocytes enhance autophagy and undergo autophagic cell death, whereas lymphocytes apoptose. Fibrinoid necrosis leads to autophagic cell death.

To investigate antigen retrieval (AR), we heated archival formalin-fixed paraffin-embedded RA synovial specimen-deparaffinized sections in EDTA solution with pH > 9 (S3307, Antigen Retrieval Reagent, high pH, Dako) for 5 min at 120 °C by autoclaving (heating-AR at high pH), and utilized an extremely sensitive indirect enzyme-labeled antibody method, the horse radish peroxidase (HRP)-labeled and secondary antibody-labeled polymer reagent method (polymer method, ordinary-IHC in Table 1) employing anti-cleaved caspase-3 antibody (Table 1). This resulted in faint labeling degenerative lymphocytes (Figure 1e). The hyperplastic and degenerative synoviocytes and the necrotic fibrinoid tissue were not labeled by anti-cleaved caspase-3 antibody when nuclear melting due to heating-AR at high pH was markedly observed in the synoviocytes (Figure 1b, e and h). After treating sections with proteinase K solution during AR (enzymatic-AR), new simplified catalyzed signal amplification (CSA) system (nsCSA system, ultra-super sensitive IHC in Table 1) [18] using anti-beclin-1 antibody (Table 1) [14,15,16]showed obvious granular immunostaining in the hyperplastic tissue, and the staining was evident in slightly degenerated synoviocytes and necrotic tissue (Figure 1c, f and i). This was likely to be due to beclin-1 forming an autophagic v esicle nucleation complex with Atg14, Vps34 and Vps15 [7].

In RA synovial tissue, lymphocytes undergo apoptosis, whereas synoviocytes enhance autophagy [19] and eventually undergo ACD through fibrinoid necrosis.

## 3. Homeostatic Mass Control of the Gastric Mucosa with and Without HP Infestation

Growth and cell death of gastric fundic glandular epithelia play a role in homeostatic mass control of the gastric mucosa. From the neck portion, gastric fundic glandular epithelial stem cells proliferate and migrate upward through a pipe line system to the foveolar epithelia and downward through a stochastic system to the gastric fundic glandular epithelia [20].

As reported previously [14], in the normal-appearing gastric mucosa of a stomach that underwent resection for cancer, a few foveolar glandular epithelia undergo apoptosis at the mucosal surface, indicated by immunostaining with anti-cleaved caspase-3 antibody. In the deep portion of the mucosa, fundic glandular epithelia showed no cleaved caspase-3^+^ apoptotic cells and few acini of beclin-1^+^ fundic glandular epithelia, suggesting that enhanced autophagy eventually led to ACD. In the inflamed gastric mucosa with HP infestation, cleaved caspase-3^+^ apoptotic cells aggregate just under the mucosal surface and appear in the neck portion, indicating anti-apoptotic effects on the surface foveolar epithelia with HP infection [21] and apoptosis-mediated removal of disordered neck cells under HP infestation [14]. 

**Figure 2:**
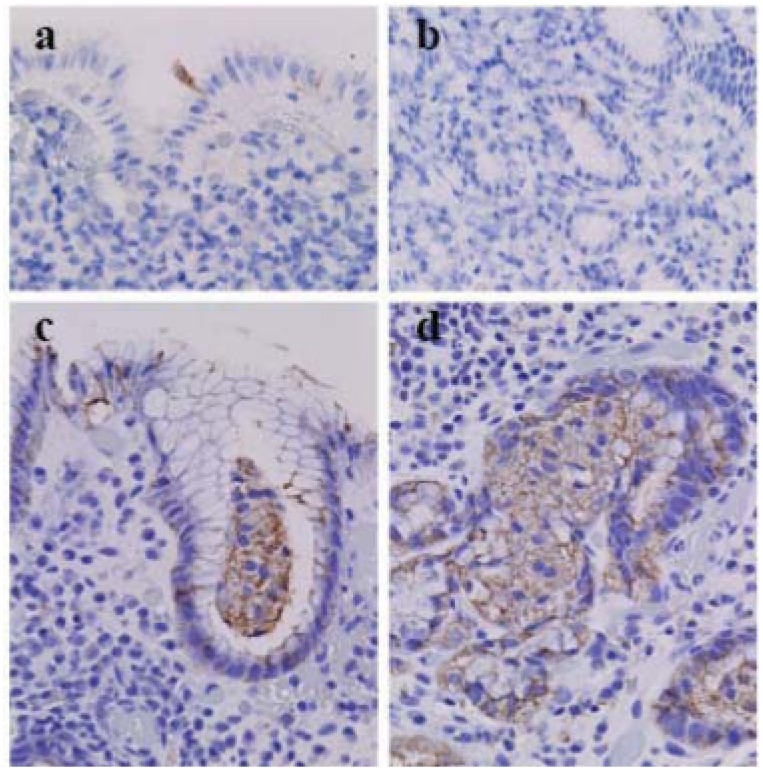
Cleaved casspase-3 and beclin-1 in the biopsied gastric mucosa with Helicobacter pylori (HP) infestation (×40, Olympus BX50, FUJIFILM HC-300). (**a**) Cleaved caspase-3^+^ foveolar epithelia in the surface of the gastric mucosa without HP infestation. (**b**) Cleaved caspase-3^+^ neck cells in the gastric mucosa with HP infestation. (**c**) A lump of beclin-1^+^ glandular epithelia in the foveolar glandular lumen of the surface portion of the gastric mucosa with HP infestation. (**d**) A group of beclin-1^+^ glandular epithelia in a gland in the deep portion of the gastric mucosa of the same case.

In the biopsied gastric fundic mucosa with and without HP infestation, cleaved caspase-3^+^ apoptotic foveolar cells are observed in the mucosal surface (Figure 2a) or just under the surface mucosa and in the neck portion (Figure 2b) [14]. However, most foveolar glandular epithelia and fundic glandular epithelia show variable granular beclin-1 immunostaining (Figure 2c and d), whereas many acini of fundic glandular epithelia show increased granular beclin-1 immunostaining (Figure 2d). A lump of glandular cells in the glandular duct, usually believed to be an artifact of specimen preparation, demonstrated increased immunostaining with anti-beclin-1 antibody (Figure 2c), suggesting that an acinus of fundic glandular epithelia underwent massive ACD and detachment from the stromal basement into the glandular lumen. 

Enhanced autophagy detection through the enzymatic-AR and nsCSA system using beclin-1 in the foveolar glandular epithelia of the biopsied gastric mucosa suggests suppression of autophagy in the stomach resected after pre-operative treatment medication such as fasting with intravenous nutrition drip [14] and persistent autophagy in the stomach without specific treatment. In homeostatic conditions some fundic glandular epithelia undergo ACD and detachment into the glandular lumen, and some new fundic glandular epithelia occupy the space in the glandular acinus, however, in inflamed gastric mucosa the space left by the detached acini of the fundic glandular epithelia may be replaced with fibrotic tissue, often observed in chronic atrophic gastritis. 

## 4. Signal Transduction in Apoptosis, Autophagy and Oxidative Stresses and Cell Death in Nasal NK/T-Cell Lymphomas (NKTCL)

**Figure 3:**
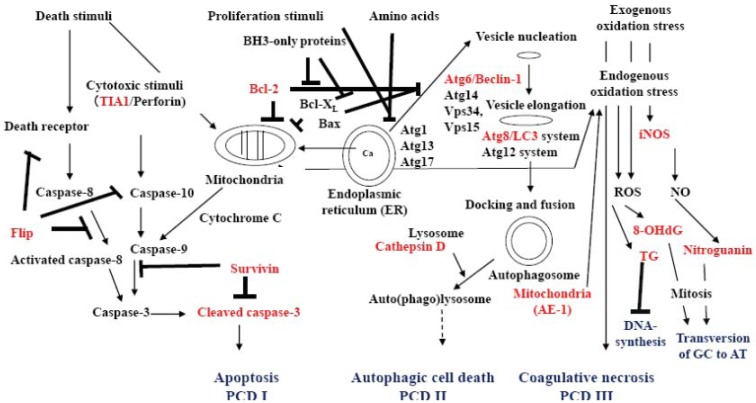
Conceptual graphic understanding of signal transduction in apoptosis, autophagy, oxidative stress and programmed cell death. Death signals from cell membrane receptors, receptor caspase-10 and from the mitochondria result in the formation of cleaved caspase-3, which initiates the signaling processes of apoptosis (PCD I). However, there are several antagonists of apoptosis, such as Flip against caspase-8, Bcl-2, Bcl-XL and Bax for mitochondria membrane stability, and survivin that is an inhibitor of apoptosis-1 (IAP-1) for cleaved caspase-3. Survivin is expressed in stem cells, fetal tissue, cancer cells and lymphoma cells. Proliferation signals and starvation (amino acid) through mTOR initiate complex formation of Atg1, Atg13 and Atg17 at the endoplasmic reticulum. Stabilization of ATG6/beclin-1 by Bcl-2, Bcl-XL and Bax is lost. Beclin-1 then forms an autophagic vesicle nucleation complex with Atg14, Vps34, and Vps15 and enhances autophagy. The Atg8/LC3 and Atg12 systems elongate autophagic vesicle to engulf a part of the cytoplasm with organelles such as mitochondria and create autophagosomes. Membrane-boundary form LC3 (LC3-II) localizes within the autophagic vesicle’s double membrane. Autophagosome fuses with a lysosome to form an auto(phago)lysosome, and the engulfed content is digested by lysosomal enzymes such as cathepsin D. Autophagy results in the recruitment of amino acids or cell survival. Over-enhanced autophagy and mal-digestion in the autolysosome lead to accumulation of autolysosomes in the cytoplasm and the cell undergoes autophagic cell death (PCD II). Strong death stimuli and exogenous and endogenous stress induce mitochondria to produce hydroperoxyl radical and metal hydroperoxo complexes, which oxidize the cytoplasm strongly and induces coagulative necrosis, possibly PCD III, and produce reactive oxygen species (ROS). In auto(phago)lysosome, mal-digested mitochondria yield endogenous ROS. Exogenous and endogenous ROS oxidizes DNA to yield 8-hydroxydeoxyguanosine (8-OHdG) and thymidine glycol (TG). However, strong stress causes the cell to express inducible nitric oxide synthase (iNOS), which generates nitric oxide (NO) and nitrates DNA to yield nitroguanosine (NG). Accumulated TG disturbs DNA synthesis. 8-OHdG and NG possibly induce GC to AT transversion in DNA when most of the 8-OHdG is removed from the repaired DNA into urine. A small amount of NO induces heam oxygenase as a signal molecule to produce an anti-oxidation response.

Nasal NKTCL is an EBV-related neoplasm [22] of CD3ε^+^ TIA1^+^ CD56^+/−^ NK or cytotoxic T cells [23], indicating peculiar necrosis that is thought to represent ACD [15,16], however it has been suggested that it may possibly represent cytotoxic granule-leakage-induced apoptosis [24]. There may be cases of NKTCL resistance to chemotherapy via enhanced antagonism of caspase-8 (Flice (caspase-8) inhibitory protein (Flip), Table 1) [25]. As shown in Figure 3, we developed an immunohistochemical analysis technique for detecting molecules involved in signal transduction in apoptosis, autophagy, oxidative stress and PCD. 

The representative molecules involved in signal transduction during apoptosis, autophagy, and oxidative stress (Figure 3) such as Flip, TIA1, Bcl-2, cleaved caspase-3, beclin-1, LC3, mitochondrial AE-1, Cathepsin D, 8-OHdG, thymidine glycol (TG), inducible nitric oxide synthase (iNOS) and nitroguanosine (NG) can be visualized using AR-IHC (Table 1). 

**Figure 4:**
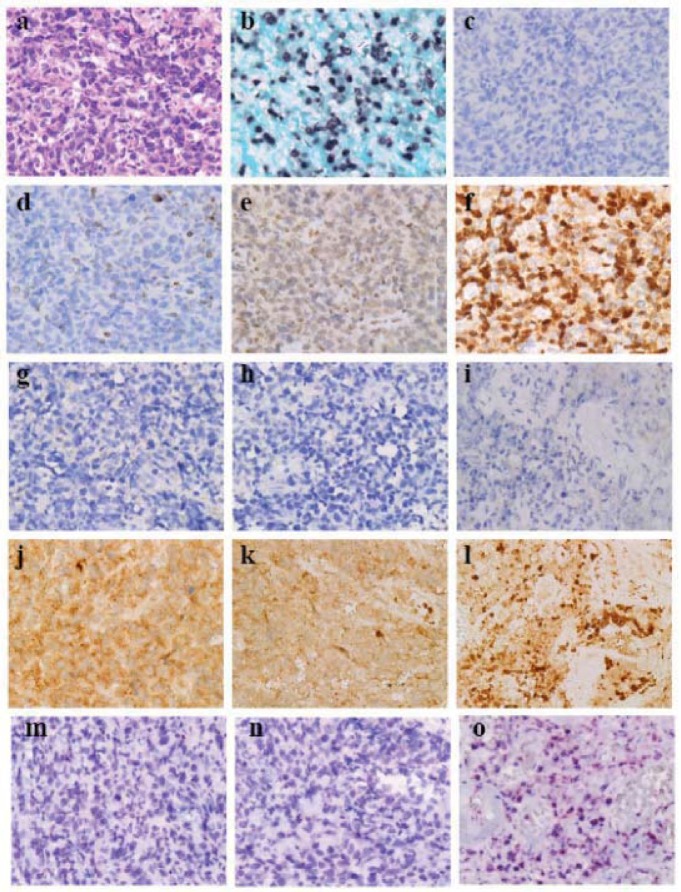
Programmed cell death (PCD)-related molecules in a case of Epstein-Barr virus (EBV)-related cytotoxic T-cell lymphoma (×40, Olympus BX50, FUJIFILM HC-300). (**a**) Cellular area (Hematoxylin-eosin staining). (**b**) In situ hybridization of EBV-encoded small RNA-1 (EBER-1). (**c**) Cleaved caspase-3. (**d**) Bcl-2. (**e**) Flice (caspase-8) inhibitory protein (Flip). (**f**) Survivin. (**g–i**) Beclin-1. (**j–l**) LC3. (**m–o**) 8-hydroxydeoxyguanosine (8-OHdG). (**g, j and m**) Cellular area. (**h, k and n**) Degenerative area. (**i, l and o**) Necrotic area. Diffuse proliferation of lymphoma cells is observed with sinusoidal patterning (a). Most lymphoma cells show EBER-1 signals in their nuclei (b). There are no cleaved caspase-3-positive lymphoma cells (c), and the lymphoma cells express Bcl-2 scantly (d), Flip moderately (e) and survivin strongly (f), suggesting that neoplastic expression of survivin suppresses cleaved caspase-3. From the cellular area to the necrotic area in peculiar necrosis, lymphoma cells do not express beclin-1-autophagic vesicle nucleation complex (g–i) but show enhanced autophagy labeled by LC3. Lymphoma cells exhibit macrogranular staining of LC3 with sporadic distribution of LC3-densely labeled cell debris or naked nuclei in the cellular (j) and degenerative areas (k) and aggregation of LC3-densely labeled cell-debris in the necrotic area (l). In the necrotic area the cell-debris is positive for 8-OHdG (o).

EBV-related cytotoxic T cell NKTCL [23] exhibits cellular, degenerative and peculiar necrotic areas [15,16]. Lymphoma cells exhibits diffuse proliferation with some sinusoid patterning (Figure 4a) [26] and indicate EBER-1 signaling in nuclei (Figure 4b). With regard to AR, after heating sections by autoclaving in Diva Decloaker (Biocare Medical) independent of the pH of the AR solution for 5 min (heating-AR independent of pH, Table 1), there were no cleaved caspase-3^+^ cells evident (Figure 4c) whereas lymphoma cells expressed scant expression of Bcl-2 (Figure 4d), moderate expression of Flip (Figure 4e) and strong expression of survivin (Figure 4f) [15], suggesting that neoplastic expression of survivin suppresses cleaved caspase-3. From the cellular area to the necrotic area in the peculiarly necrotic tissue, lymphoma cells do not express beclin-1 (Figure 4g–i) but show enhanced autophagy when labeled by LC3 [15,16]. Lymphoma cells exhibit macrogranular staining of LC3 with sporadic distribution of LC3-densely labeled cell-debris or naked nuclei in the cellular (Figure 4j) [17] and degenerative areas (Figure 4k) and show aggregation of LC3-densely labeled cell debris or naked nuclei in the necrotic area (Figure 4l) [15,16]. No obvious labeling of beclin-1-autophagic vesicle nucleation complex and enhanced autophagy labeled by LC3 suggest that certain signals may be present that accelerate autophagy after autophagic vesicle nucleation [16]. Through heating-AR independent of pH and alkaline phosphatase-labeled and secondary antibody-labeled polymer reagent methods (ordinary-IHC* in Table 1) for anti-8-OHdG antibody (Table 1) labeling, 8-OHdG was labeled in the cell-debris in the necrotic area (Figure 4o), suggesting that mal-digested mitochondria in autolysosomes yield reactive oxygen species (ROS) that oxidize DNA. 

## 5. Immunohistochemistry of Programmed Cell Death

From our above-mentioned and limited experiences of AR-IHC, cleaved caspase-3, survivin, beclin-1, LC-3 and 8-OHdG are believed to be the target molecules for differentiating apoptosis (PCD I), ACD (PCD II) and coagulation necrosis (PCD III) in normal cells under homeostatic mass control and in neoplastic cells (Figure 3). 

Cleaved caspase-3 can be detected by using a polymer method involving heating-AR at high pH or heating-AR independent of pH. Since nuclear melting is occasionally observed when heating-AR at high pH (Figure 1b, e and h), the suitable AR-IHC techniques for detecting cleaved caspase-3 includes heating AR independent of pH and polymer method. In the gastric fundic glandular epithelia, stem cells differentiate into foveolar glandular epithelia that undergo apoptosis at the mucosal surface, or into fundic glandular epithelia, such as chief cells and parietal cells, that do not express cleaved caspase-3 and undergo ACD. The fundic glandular epithelia presumably acquire an unknown anti-apoptotic mechanism during their differentiation. One such anti-apoptotic mechanism in the fundic glandular epithelia is probably the induction of Flip as in macrophages and synoviocytes [17], however, a representative anti-apoptotic mechanism in cancer cells and lymphoma cells is the induction of survivin [27,28]. The expression of both Flip and survivin can be visualized through the heating-AR independent of pH and the polymer method [15]. 

Low expressions of beclin-1 cannot be detected by usual sensitive methods such as the polymer method, but can be visualized through ultra-super sensitive IHC, which is 1,000 times more sensitive [14]. Heating-AR at pH 6 and using the nsCSA system of beclin-1 labels most cells [14], suggesting that beclin-1 stabilized with Bcl-2/Bcl-X [9] is more abundant than that forming an autophagic vesicle nucleation complex with Atg14, Vps34 and Vps15 [9]. Enzymatic AR and the nsCSA system can label beclin-1, probably in the autophagy vesicle nucleation complex with Atg14, Vps34 and Vps15 [9], enabling visualization of enhanced autophagy [14]. Fibrinoid degeneration in RA exhibited massive deposition of beclin-1 in the necrotic tissue, suggesting that fibrinoid degeneration in RA represents massive ACD [16]. However, the expression of beclin-1 detected through the enzymatic-AR and nsCSA system decreased according to the degeneration to necrosis of NKTCL when enhanced autophagy labeled by LC3 [16], as mentioned above. After sufficient autophagic vesicles are generated, rapid elongation of the autophagophore labeled by the LC3 probably induces ACD; however, this mechanism is not known. Dense labeling of the LC3 in naked nucleus-like cell-debris is a hallmark of ACD [15,16] because autophagosomes and auto(phago)lysosomes with membrane-boundary LC3 (LC3-II) populate the cytoplasm of cells undergoing ACD. Therefore, there are at least 2 enhancing pathways of autophagy: the ordinary pathway with increased beclin-1-vesicle nucleation complexes and the “peculiar” pathway with enhanced autophagy labeled by LC3 without increased beclin-1-vesicle nucleation complexes. The former is detected in non-neoplastic synovial tissue and the latter in EBV-related NKTCL. The peculiar pathway may be a feature of neoplastic cells, although latent EBV infection may play a role in EBV-related NKTCL [15,16].

As for the IHC of anti-LC3 antibody (PM036/MBL) with AR independent from the pH of AR solution [16,17], it was reported that “Since the immunostaining of the membrane-boundary form LC3-II reflects the autophagic vesicle elongation, the specific evaluation of LC3 must be done in the background immunostaining of LC3-I” [15]. Therefore, granular immunostaining of anti-LC3 antibody (PM036/MBL) could be evaluated as that of LC3-II. As reported [16], the dominant immunostaining of AR-IHC of LC3 is evaluated in 6 scores; score 0 (no staining), score 1 (microgranular staining in cytoplasm), score 2 (macrogranular staining in background of strong microgranular staining in cytoplasm), score 3 (macrogranular staining in background of decreased microgranular staining in cytoplasm), score 4 (nuclear or perinuclear dense staining in background of scores 2 and 3) and score 5 (nuclear or perinuclear dense staining without the background of scores 2 and 3. The scores 4 and 5 correspond to regional ACD [16] (Figure 4l), as mentioned above. Scores 1 to 3 would correspond to physiological and enhanced autophagy as a prosurvival response, and associate sporadic ACD (Figure 4j) that is a surrogate for apoptosis.

Mitochondria are representative organelles for macroautophagy. Cathepsin D is one of the lysosome enzymes that digest contents in the autolysosome. Comparative AR-IHC of mitochondria and cathepsin D can monitor progression of autophagy [16]. Aggregated mitochondria with low cathepsin D expression in NKTCL cells during degeneration before massive ACD suggested that low synthesis of cathepsin D induces massive ACD in NKTCL [16], as reported by Uchiyama [8]. The mechanism of specific engulfment of cytoplasm (microautophagy and chaperone- autophagy) and organella (macroautophagy) and specific docking with lysosome and with endosome for inducing innate immunity would be the next molecular and cell biological investigation targets in autophagy. After specific molecules and their functioning forms in these aspects of autophagy are elucidated, AR-IHC of those molecules is expected to be introduced in monitoring autophagy in human pathology specimens since intercalated antibody polymer method (Dako EnVision^TM^ FLEX) and ultra-sensitive IHC through CARD reaction (Dako CSA II system, new simplified CSA system [18]) are ready to be employed for AR-IHC of such target molecules.

8-OHdG, a well-known marker of oxidative stress, is usually excreted into the urine following DNA repair. As shown in Figure 4, immunostaining of 8-OHdG in the cell-debris in the necrotic area of NKTCL suggested that endogenous ROS, probably from mitochondria in auto(phago)lysosomes [29], oxidized DNA in the cell-debris. Cell death under conditions of strong oxidative stress, such as that induced by hydroperoxyl radical and metal hydroperoxo complexes, probably oxidizes the cytoplasm in coagulative necrosis/PCD III, although this remains to be investigated. In such cell death, nuclear DNA would be oxidized and 8-OHdG would appear in the DNA. Consequently, coagulative necrosis/PCD III could be detected by heating-AR independent of pH and alkaline phosphatase-labeled and secondary antibody-labeled polymer reagent methods, thus preventing synthesis of 8-OHdG in inactivating endogenous peroxidase in peroxide methanol or phosphate buffered saline solution. On the other hand, enhanced autophagy evokes antioxidant responses through p62 [30]. Strong non-specific stress induces iNOS in variable cells and iNOS supplies nitric oxide (NO). Sufficient NO nitrates guanosine to NG, whereas low NO plays as signal molecules to induce antioxidant responses such as the induction of heam oxygenase-1 [31]. In addition to the above-mentioned AR-IHC for autophagy, comparative AR-IHC of 8-OHdG, TG, iNOS and NG (Table 1) may evaluate oxidative stresses and antioxidant responses in archival pathology specimens when that of TG reflects accumulation of oxidative stresses.

The use of IHC for detecting PCD in the archival human pathology specimens is expected to extend to the field of apoptosis, autophagy and DNA oxidation and nitration. Characterization of the differences between the signal molecules involved in the PCD of neoplastic and non-neoplastic cells might be useful in the differential diagnosis of neoplastic cells in surgical pathology as well as AR-IHC of survivin [15]. Further, the IHC of PCD may offer a range of novel target molecules involved in autophagy in human neoplasia and lead to the production of new anti-neoplasia drugs.
